# Acceptability and Utilization of Three Nutritional Supplements during Pregnancy: Findings from a Longitudinal, Mixed-Methods Study in Niger

**DOI:** 10.3390/nu10081073

**Published:** 2018-08-12

**Authors:** Adrienne Clermont, Stephen R. Kodish, Amadou Matar Seck, Aichatou Salifou, Joseph Rosen, Rebecca F. Grais, Sheila Isanaka

**Affiliations:** 1 Department of International Health, Johns Hopkins Bloomberg School of Public Health, Baltimore, MD 21205, USA; aclermo2@jhu.edu (A.C.); jrosen72@jhu.edu (J.R.); 2 Departments of Nutritional Sciences and Biobehavioral Health, Pennsylvania State University, University Park, PA 16802, USA; quk80@psu.edu; 3 Epicentre Niger, BP, 465 Maradi, Niger; Amadou.MATAR@epicentre.msf.org (A.M.S.); Aichatou.SALIFOU@epicentre.msf.org (A.S.); 4 Department of Research, Epicentre, 75012 Paris, France; Rebecca.GRAIS@epicentre.msf.org; 5 Departments of Nutrition and Global Health and Population, Harvard T.H. Chan School of Public Health, Boston, MA 02115, USA

**Keywords:** maternal nutrition, pregnancy outcomes, prenatal supplement, Niger, diet, qualitative methods

## Abstract

Nutritional status in pregnancy is a key determinant of birth outcomes. In low-income countries, maternal diets are often limited, and daily nutrient supplements are recommended to fill nutrient gaps. As a result, it is important to understand the factors influencing acceptability and utilization of nutrient supplements in these settings. Qualitative data (individual interviews and focus group discussions with pregnant women, household members, and study staff) and quantitative data (unannounced household spot checks) were collected in 24 villages in the Maradi region of south-central Niger. Each village was randomly assigned to one of three study arms, with pregnant women receiving either iron and folic acid (IFA) supplements, multiple micronutrient (MMN) supplements, or medium-quantity lipid-based nutrient supplements (MQ-LNS) for daily consumption during pregnancy. Data were collected longitudinally to capture changes in perspective as women progressed through their pregnancy. Participants accepted all three supplement types, and perceived a wide range of health benefits attributed to supplement consumption. However, several important barriers to appropriate consumption were reported, and rumors about the supplements leading to childbirth complications also decreased utilization. The household spot checks suggested that IFA had the highest level of correct consumption. Overall, despite a stated high level of acceptance and enthusiasm for the supplements among participants and their household members, certain fears, side effects, and organoleptic factors led to decreased utilization. The effectiveness of future programs to improve maternal nutritional status through supplementation may be improved by understanding perceived barriers and facilitating factors among participants and tailoring communication efforts appropriately.

## 1. Introduction

Adequate nutrition in pregnancy is essential for maternal health and positive birth outcomes [1,2]. However, traditional diets in many low-income countries are suboptimal, and additional individual and socio-cultural factors, such as food aversions and “eating down” in pregnancy [3,4,5,6], can further reduce intake in pregnancy. Nutrient supplements can help fill this gap and may improve pregnancy outcomes [1,7]. The World Health Organization (WHO) currently recommends daily iron and folic acid (IFA) supplementation in pregnancy [8], but coverage in most countries remains low. In 2012, only 28.6% of women in Niger reported consuming iron for 90 or more days during their most recent pregnancy [9]. Multiple micronutrient (MMN) supplements have been shown to be more effective than IFA alone on improving birth outcomes [10], but it is not yet recommended by the WHO as the standard of care [8] and utilization remains low. Lipid-based nutrient supplements (LNS) are another emerging option for prenatal nutritional supplementation, providing protein and energy as well as micronutrients, but are rarely used outside of experimental contexts [1]. All three supplements must be consumed on a daily basis throughout pregnancy to optimize effectiveness; as a result, understanding factors influencing acceptability and utilization of these supplements by pregnant women remains a crucial component to improving maternal health outcomes.

Previous qualitative and mixed-methods studies have been conducted on the acceptability and utilization of IFA [11,12,13,14,15,16,17], MMN [18,19,20,21], and LNS [16,22,23] in community settings in low-income countries. Measured levels of adherence to supplementation protocol have varied widely in these studies, from 95% in Mali [18] to 47% in Cambodia [12]. A large number of individual and program-related factors have been identified as influencing adherence, including organoleptic properties of the supplements [13,16,20,22], provision of the supplement at no cost [14,21], reminders to take the supplement [15,16,21], and perceptions of supplement benefits [14,15,17,20]. Fewer studies have examined household- and community-level factors affecting participants, or have allowed for a comparison across all three supplement types (IFA, MMN, and LNS) in the same study context.

The objective of this paper is to examine the factors influencing acceptability and utilization of these three supplements among a rural population in southern Niger using a longitudinal, mixed-methods design. In this paper, we discuss the typical consumption, perceived benefits, facilitating factors, and barriers to appropriate utilization reported by participants for each of the three supplement types over the course of pregnancy. We also examine household and community member perceptions of supplement utilization and triangulate qualitative findings with quantitative household utilization data from unannounced spot checks.

## 2. Materials and Methods

### 2.1. Study Site and Population

This study was conducted within an ongoing randomized trial designed to assess the effectiveness of three prenatal supplements to improve infant immune response to rotavirus vaccination (ClinicalTrials.gov Identifier: NCT02145000) [24]. The study took place in the Madarounfa Health District within Maradi region, a rural area in south-central Niger along the Nigerian border. Nutritional outcomes in the region are poor. According to the 2012 Demographic and Health Survey (DHS), 17.9% of women of reproductive age were underweight (body-mass index of <18.5 kg/m^2^) and 53.5% of children under five were stunted (<−2 *z*-score in height-for-age), both of which were worse than the national average rates for Niger [9].

Villages in the parent study (*n* = 53) were randomly assigned in a 1:1:1 allocation to one of the three study arms (IFA, MMN, or LNS). Pregnancy surveillance was conducted among all consenting non-pregnant women of reproductive age, and women who became pregnant and met inclusion criteria were enrolled to receive supplementation until the end of their pregnancy (approximately 6 months). Participants meeting inclusion criteria were less than 30 weeks gestation at the time of enrollment; intended to remain in the study area through delivery and for 2 years thereafter; and did not have a chronic health condition, severe illness, evident pregnancy complications, or peanut allergy. All participants received free antenatal care provided by the study as per the national protocol.

A mixed-methods sub-study to assess acceptability and utilization of the supplements included pregnant women, as well as household and community members, from 24 villages participating in the parent study. These villages, and the specific participants selected for data collection, were selected using criterion-based purposive sampling [25] in order to gather a representative sample of participants’ views, based on pre-established criteria that were believed to be important based on the literature (including maternal age, gestational age, and supplement type). Sample size ranges were chosen based on estimates of the number of participants needed to reach data saturation [26].

Written informed consent was obtained from all study participants (as well as their husband or father if the participant was less than 18 years of age). The study was approved by the Comité Consultatif National d’Ethique in Niger; the Comité de Protection des Personnes in France; the Commission d’Ethique de la Recherche sur l’Etre Humain, Hôpitaux Universitaires de Genève in Switzerland; Research Ethics Review Committee of the World Health Organization in Switzerland; and the Western Institutional Review Board in Olympia, WA.

### 2.2. Study Intervention

The IFA group received tablets containing 60 mg iron and 400 µg folic acid, according to the national guidelines of Niger. The tablets were red and came in a blister pack of 10 tablets. Participants were instructed to take one tablet daily, with water after the evening meal. The MMN group received gel capsules containing a white powder, with a daily dose (four capsules) containing 30 mg iron, 400 µg folic acid, and 20 other micronutrients. They received a plastic bottle containing 40 capsules and were instructed to take two in the morning and two in the evening, with water and after meals. The LNS group received 40 g of a fortified, ready-to-use food made of peanuts, oil, dried skimmed milk powder, sugar, and the same 22 micronutrients as the MMN group. Because of its size, this product was classified as a medium-quantity LNS (MQ-LNS) and will henceforth be referred to as such [27]. The MQ-LNS group received 10 individual foil sachets of MQ-LNS, and were instructed to take one a day, either alone or mixed with already prepared family foods. The detailed nutritional composition of the supplements is available in Table S1. 

The supplements and instructions for use and household storage were presented by a study midwife at the time of enrollment. Health assistants conducted weekly home visits to distribute a 10-day supply of the supplement (7 days’ supply to be consumed, plus 3 days’ extra supply in case of loss or damage, otherwise to be returned to the health assistant at the next distribution). At the weekly home visits, the health assistants reviewed supplement adherence and discussed other health events or concerns with the study participants. 

### 2.3. Study Design and Data Collection

This was a two-phase, longitudinal study with emergent design, in which Phase 2 data collection built off findings from Phase 1. We collected quantitative and qualitative data to compare the acceptability and utilization of the three supplement types, as well as to understand the factors influencing acceptability and utilization among participants. The number of interviews, focus groups, and spot checks conducted by participant type, study arm, and study phase are shown in Table 1. 

Qualitative and spot check data were collected by study staff who were trained in qualitative research methods and not involved in supplement distribution. Semi-structured interview and focus group guides covered supplement acceptability, consumption practices, facilitating factors and barriers, perceived side effects, perceived benefits, support or opposition from household members (e.g., husbands and in-laws), and the supplement delivery mechanism. In anticipation of the sensitive nature of the questions, or the potential for respondents to hesitate to share negative comments or practices, the interviewers were trained to ask respondents to share experiences of other women that they might be aware of, as well as their own experiences. Data were collected in two different phases (July–August 2016 and November–December 2016) to allow for a longitudinal analysis that would capture changes in perspective as women progressed through their pregnancy and gained experience with the supplements. Interviewers were also asked to give a numerical Likert scale rating of both acceptability and utilization (each on a 0 to 10 scale, with 10 being the best) with textual field notes for further triangulation of data from the interviewers’ perspective. 

In Phase 1, semi-structured individual interviews and focus groups were conducted with participating women at various gestational ages (*n* = 84 interviews and 17 focus groups), their husbands (*n* = 18 interviews) and mothers-in-law (*n* = 9 interviews), and study staff (midwives, *n* = 3 interviewers, and health assistants, *n* = 9 focus groups). Women in their first, second, and third trimesters were purposively sampled in Phase 1. In Phase 2, individual interviews and focus groups were conducted with participating women in the third trimester of their pregnancy or who had recently delivered (*n* = 39 interviews and 12 focus groups). These were re-interviews of women who had participated in Phase 1, in order to generate an understanding of change in women’s attitudes and experiences with supplement consumption over the course of the pregnancy.

Household spot checks were conducted in Phase 1 (*n* = 90) and Phase 2 (*n* = 50) in order to count the number of supplements on hand and to calculate supplement adherence. Spot checks were unannounced and conducted after the completion of an interview by asking to see the household stock of supplements. A convenience sampling method was used for spot checks, from among participants already selected for interviews or focus groups.

### 2.4. Data Analysis

Interviews and focus groups were conducted in the local language (Hausa), then transcribed verbatim and translated into English. The English transcripts were uploaded into Dedoose software [28] for data management and analysis. The transcripts were coded using an inductive approach, drawing from aspects of Grounded Theory [29,30,31]. In this approach, rather than using a pre-determined codebook, an exhaustive list of codes was established through the analysis of participant transcripts. These codes were subsequently grouped into emergent themes and categories in order to determine the most salient findings and the frequency with which they appeared in each of the three study arms. In addition, coded text was displayed in a thematic matrix and stratified by study arm, gestational age, and interviewer ratings for enhanced pattern identification [32].

For quantitative analysis of the spot check data, the expected number of supplements was calculated based on the number of days elapsed since the last distribution, which was then compared to the actual number observed during the spot check. For the purposes of this analysis, correct consumption was defined as the actual number being no more than 2 days’ worth of supplements above or below the expected number (i.e., within two tablets or sachets for IFA and MQ-LNS, and within eight capsules for MMN). If the actual number exceeded the expected, this was considered “under-consumption”, and if the actual number was less than expected, this was considered “over-consumption”.

## 3. Results

The main themes from the interviews and focus groups, along with their relative frequency by study arm, are summarized in Table 2. They are discussed in further detail, with inclusion of exemplar quotes from respondents and interviewer field notes below.

### 3.1. Typical Utilization

Women did not report significant problems with IFA consumption; they typically swallowed it with water or with *fura* (a millet-based porridge mixed with milk). Most women took the tablet in the morning after their breakfast, although some took it in the evening to avoid morning sickness early in pregnancy.

Since she was given this supplement, she takes it easily … and swallows it with water. She does not have any fear of taking it.

*(Interviewer field notes, IFA group, Phase 1, ID#52)*

The MMN supplement consisted of a white powder inside a gel capsule. While some women were able to swallow the capsule comfortably with water, others chose instead to open it and mix the micronutrient powder inside with water or with food (*fura* or other porridge) in order to consume it.

Early in the morning after I take my breakfast I will open the capsule of the supplement and take two supplements, and likewise in the evening … We have heard the layer that covers the supplement does not dissolve in the body; that is why I remove it before I take the supplement … We are removing the capsule then pouring the solid medicine into water for consuming.

*(Pregnant woman, MMN group, Phase 2, ID#47)*

There was a strong consensus that the IFA and MMN supplements should not be taken on an empty stomach. This appeared to be due to instructions from the study team at supplement distribution that advised taking it with food, as well as general beliefs around the consumption of other medications (e.g., malaria treatment).

Most women chose to consume the MQ-LNS alone, or less commonly to mix it with porridge. Some women mixed it with sugar or with other foods in order to avoid the taste of the MQ-LNS.

Since the time that (the health assistant) gave me the supplement, I spent two weeks vomiting whenever I used to take it. Later, I complained to him and he told me that I should be taking it with fura and put sugar inside it, or I should be taking it just like that and I will become used to it. That is how I have done and now I am enjoying it.

*(Pregnant woman, MQ-LNS group, Phase 1, ID#86)*

### 3.2. Perceived Benefits

Participants in all three groups most commonly reported increased appetite during pregnancy, more strength and energy to do household chores, and overall improved health (prevention of pregnancy illnesses) as the most important benefits to themselves. Consumption was also perceived to lead to safe delivery of a healthy baby, which would be evident from the baby’s large size (frequently contrasted with small, “malnourished” babies), good complexion and smooth skin, and absence of diseases such as diarrhea and fever. These findings were consistent across all three study arms.

It promotes and protects my health, protects my growing fetus, increases my appetite, makes me strong, limits the risk of developing minor infections and diseases, enhances safe delivery, and prevents shortage of blood in the body.

*(Pregnant woman, IFA group, Phase 1, ID#68)*

Before, I couldn’t do any work; other people had to do it for me. But since I started consuming this supplement, by God’s grace, I can cook, sweep, bathe my children, and do all the minor housework without getting tired. This supplement is giving me strength and energy; it also builds up my body and the baby in the womb.

*(Pregnant woman, MMN group, Phase 1, ID#88)*

Particularly in the IFA group, curing anemia and “increasing blood volume” in the body were also cited as important benefits. Many participants recognized the “red pill” as being associated with blood, due to having received iron tablets from healthcare providers in previous pregnancies. Increased blood was seen as important for a healthy delivery.

When a woman is using the supplement, it increases the volume of blood in her body, so that when she delivers she will not need any blood transfusion because she has enough blood, even after delivery.

*(Pregnant woman, IFA group, Phase 1, ID#62)*

### 3.3. Facilitating Factors

Beyond the supplements themselves, a high level of trust in doctors and the public health system in general was reported by study participants, which was further bolstered by the provision of free healthcare (from antenatal care to the baby’s second birthday) by the parent study. This appeared to be a facilitating factor in encouraging supplement adherence.

We don’t know the actual benefits (the supplement) will provide to us, but we are totally sure it will do something good in our body, because whatever comes from the hand of a doctor must be essential to life.

*(Pregnant woman, MMN group, Phase 2, ID#45)*

Many participants reported that seeing healthy babies delivered by other women in the community who were using the supplements was a powerful motivating factor to enroll in the study and continue adherence.

Yes, the supplement is easy to consume. Also, it makes a delivery easier for the pregnant woman that consumes it. For instance, my neighbor gave birth to a baby girl two weeks ago. The baby looks very big and healthy. The mother also told me that she delivered the baby in a very short period of time and she thinks it is the supplement that made it easier for her to deliver. This is one of the things that encourages me to consume the supplement every day.

*(Pregnant woman, IFA group, Phase 1, ID#93)*

### 3.4. Acceptability-Related Barriers

All three groups reported organoleptic complaints. For IFA, the most common complaints were that the tablet smelled bad or was too big.

I don’t have any problem consuming the supplement, but after taking the supplement, I feel some choky thing inside my throat and I am feeling as if I want to vomit out what I took in ... After some time, it goes away and I get back to my normal state of being healthy and enjoying my day.

*(Pregnant woman, IFA group, Phase 1, ID#31)*

For MMN, the large size of the capsule was seen as a problem by many women, but the frequency of consumption (four per day) was not explicitly mentioned as a barrier. For those that opened the capsule to consume the powder with water or food, a negative odor of the powder inside was reported. For MQ-LNS, many women disliked its taste, reporting it as “bitter” or “not sweet,” while others reported enjoying the “sweetness” of the product; this seemed to be a matter of personal preference and the level of food sensitivities experienced in pregnancy. It may also have been related to past exposure to Plumpy’Nut, a ready-to-use therapeutic food used in the treatment of severe acute malnutrition among children, which is significantly sweeter than the MQ-LNS developed for adult women in this study. (Questions about experience with Plumpy’Nut and other similar products were not included in the interview guide, so the overall level of familiarity among the participant population could not be assessed.)

Self-reported side effects across all groups included vomiting, nausea, dizziness, and weakness, although at least some women speculated that these symptoms might be due to their overall pregnancy condition rather than the supplement itself.

As mentioned above, all three study arms were instructed to take the supplement with food, which sometimes led to skipping when food was not available in the house. The MMN group in particular reported not wanting to take the capsules without a meal; it is unclear why this was reported more frequently in the MMN group than the others.

Sometimes we wake up in the morning without having breakfast, so we must look for what to eat, and the drug is to be taken after eating food, so if time passes without getting something to eat, we must skip that instance (of taking the supplement).

*(Pregnant woman, MMN group, Phase 1, ID#47)*

### 3.5. Utilization-Related Barriers

Despite overall trust in the public health system, many rumors about the parent study were reported to be circulating in the study communities. These included distorted stories about the vaginal exam conducted as per the national protocol and blood samples collected as a part of the study.

At first, there are some people in this community that tried so hard to see that pregnant women didn’t use the supplement. They went on spreading rumors that the supplement will cause nothing but harm to the people of this community; they also tried to scare us off the supplement by telling us all sort of lies that the doctors use to remove some liters of blood from the body, and they also said that the doctors use to cause miscarriage for the women. They also said that it is a male doctor that does the diagnosis [including vaginal exam].

*(Pregnant woman, MMN group, Phase 1, ID#116)*

By far the most commonly cited rumor was that taking the supplements would cause the fetus to grow too big, leading to a painful delivery or complications. This was prevalent across all three study arms.

It is because the supplement makes the a baby to grow big inside the womb, and when the baby is too big, the mother will have a tear during delivery or may not be able to deliver the baby herself until she undergoes a Cesarean section.

*(Pregnant woman, IFA group, Phase 1, ID#112)*

For the IFA supplement in particular, although increased blood volume was primarily discussed positively, some women expressed fear that excessive blood in the body could lead to negative consequences (i.e., hemorrhage) during delivery.

I used to forget about the supplement … I don’t have any specific reason … But I don’t want to have excessive blood in my body, that is why I decided to reduce my consumption of the nutritional supplement.

*(Pregnant woman, IFA group, Phase 1, ID#25)*

Other women are saying about the supplement that it causes severe bleeding during childbirth, which leads some women to not consume the supplement. They may put it aside or discard it in the trash.

*(Pregnant woman, IFA group, Phase 1, ID#68)*

Taken together, fears about childbirth complications due either to an overly large baby or to excessive bleeding were the most commonly cited factor leading to non-adherence in the IFA group, more so than reported side effects or complaints about organoleptic factors such as taste or size. 

Because MQ-LNS more closely resembled a food, over-consumption of the supplement by women and their household members appeared to be prevalent. Some women reported consuming more than one sachet per day if they were hungry. Many reported sharing MQ-LNS with household members, particularly with small children who would “cry” if they saw the supplement being consumed in their presence without receiving at least a taste.

If the supplement is going to be given to me for the whole of the year, I wouldn’t mind consuming it; I even hide it from the children so that they won’t take it, because of its sweetness. If I eat it in front of them, they will be the one to consume half of the supplement, because they would say I must give it to them too. I also don’t store it where the children will see it.

*(Pregnant woman, MQ-LNS group, Phase 1, ID#4)*

It seemed that there was some level of familiarity with LNS products in the communities, as some participants referred to the supplement as “plumpy” (presumably having previously seen or tasted Plumpy’Nut). Although no participants admitted to doing so themselves, a few reported that other women in their communities sold their MQ-LNS sachets in the market for cash.

Some just take it as a business, especially the LNS, thinking that it is a nutritional supplement for malnourished children, and they sell it 25 CFA or 50 CFA [US $0.04 to $0.09]. But now they have stopped that; since we are asking them to bring the used empty sachets, we get fewer cases of this.

*(Health assistant, MQ-LNS group, Phase 1, ID#109)*

Many participants seemed unwilling to report their own non-adherence, instead preferring to focus on the perceived benefits of the supplement and thankfulness to the study for providing support. However, they appeared to feel more comfortable reporting they knew “other women” who chose not to consume the supplements, instead throwing them away or feeding them to farm animals. Mentioning the behavior of others appeared to be an important way to express lack of acceptability and poor utilization in the community.

With the exception of the fear of excessively large babies, which appeared to be widespread, many women were reluctant to admit that they themselves believed or were influenced by rumors about the supplements or the parent study. Others actively rebutted these rumors based on their own positive experiences. However, they readily acknowledged that the rumors were present in their communities.

You will see that, especially for new programs, people will be saying different things about that program for some time. In the village, people were saying that the supplement will result in the death of the baby. If they were right, I believe from the day Epicentre started this program, thousands of children would have died by now. So these are just rumors, because since I started taking the supplement I have become healthy and I have seen many other successfully delivering a healthy baby. I have not ever listened to them, because in my mind I have no doubt about the program. That is why I am taking the supplement and even taking my children for immunization, despite all different kinds of rumors I am hearing from people.

*(Pregnant woman, MMN group, Phase 1, ID#88)*

The interviewers’ Likert scale ratings of respondents’ acceptability and utilization of the supplements were generally in line with the qualitative findings from the interviews and focus groups. Interviewers rated all three supplements very similarly, with MQ-LNS rated the most highly on both the acceptability and utilization scales (mean acceptability score: MQ-LNS = 7.9, IFA = 7.6, MMN = 7.4; mean utilization score: MQ-LNS = 7.6, MMN = 7.3, IFA = 7.1). However, during the thematic matrix analysis no association was apparent between the interviewer ratings and participants’ self-reported acceptability and utilization.

### 3.6. Family, Community, and Health Staff Perceptions

Interviews with husbands and mothers-in-law were also conducted, and largely echoed the themes of the women participants. They reported the same perceived benefits, barriers to consumption, and negative rumors. Mothers-in-law had less direct knowledge of women’s daily consumption habits, but were nonetheless positive about the supplements overall.

I don’t know how they (my daughters-in-law) take the supplement. I just advise them to take it, and not to quit. Every pregnant woman should take her supplements and go for antenatal care, this is indeed a great improvement to our nation; we really appreciate the help and we enjoy how the care is given to us. The mother and the baby will remain healthy. The healthcare and the medication are also free. I really appreciate it.

*(Mother-in-law, MMN group, Phase 1, ID#14)*

Husbands were supportive of their wives’ participation, but beyond their initial granting of permission, their level of engagement varied. Some reported strongly supporting their wives’ supplement consumption and asking about it every day, while others admitted not being deeply familiar with the details.

My impression on the supplement is neutral. (My wife) went to the hospital and collected the supplement; I will not tell her to stop consuming the supplement … If there is anything wrong with the supplement, the doctors would not bring it here and ask pregnant women to consume it. But I do not know any additional information about the supplement … She could have told me she doesn’t like it, but from what I understand, my wife enjoys consuming this nutritional supplement.

*(Husband, IFA group, Phase 1, ID#64)*

Husbands particularly valued their wives remaining healthy and strong during pregnancy, a safe childbirth, and a baby free of illnesses. They often measured their perceptions of benefit in terms of a comparison to their wives’ previous childbearing experiences.

My first child did not experience this program, but my second child did. My first son had many problems when his mother gave birth to him, but my second son was born very healthy, so I came to realize that it is the supplement that his mother consumed during pregnancy that prevented those diseases from reaching him.

*(Husband, MQ-LNS group, Phase 1, ID#19)*

Women unanimously reported that their husband was the most important decision-maker in the household. Without his permission, they would not be able to take part in the study. Although some cited other women in the community whose husbands had barred them from joining, the vast majority of participants reported that their husbands were supportive and encouraged them to continue taking the supplement.

There is nobody that can stop me from taking the supplements. Even my in-laws cannot, only my husband can stop me ... and my husband likes the supplement, he even encourages me to be taking the supplement on time ... He is the one that guides me every day. I am his second wife, but even if he didn’t sleep in my room [that night], in the morning he will come to my room and ask me whether I took the supplement or not.

*(Pregnant woman, MMN group, Phase 1, ID#89)*

Conversations about the supplement with aunts, mothers, mothers-in-law, co-wives, and neighbors were also mentioned less frequently. Many of these interactions were positive, but even if they were negative, they were not cited as major factors in decision-making.

Whenever I want to take (the supplement) I have to hide so that my mother-in-law will not see me, because she doesn’t like the supplement ... She believes the rumors in town that it makes a woman not to be able to deliver the baby herself due to the big size of the baby; she has to undergo an operation. But we don’t believe this; it is not the supplement, this happens by God’s will.

*(Pregnant woman, MQ-LNS group, Phase 1, ID#105)*

Midwives working at the study health centers and health assistants who conducted the weekly supplement distributions were also interviewed during data collection. Their primary role was in explaining the health benefits of the supplements, dispelling rumors and concerns, and promoting proper consumption. They also shared recommended coping mechanisms for women who struggled to take the supplement.

The women taking IFA complain of an irritating smell, so we tell her to put it in food and take it when going to bed (to avoid morning sickness); we tell them to do such because they will not feel it and if they go to sleep nothing will happen. For those who cannot take MMN because of its size, we tell them to take off the capsule and put the powder in fura or cool porridge – not the hot one, because the hot one makes the MMN less active – and we explain to them that it is not a problem. The LNS should be mixed with water for those that cannot endure to take it the way it is, or mixed in a porridge.

*(Midwife, Phase 1, ID#81)*

The health assistants in particular had a role in sensitizing women and their families. They were well accepted by participants, who found the weekly home visits helpful and convenient. Many of the health assistants were male, and although this was initially met with resistance from husbands, it seemed to be overcome by study awareness campaigns.

The challenge we had from the initial point of the service was that some husbands were not happy because we are men going to their wives, but everything became normal after they understood what the service is. Some husbands even come to us themselves to inform us that their wives are pregnant so that we can involve them in the service, because they are happy with the project. Truly, we don’t have a problem.

*(Health assistant, MMN group, Phase 1, ID#134)*

The health assistants appeared to be convinced of the importance of the supplements, and passed this on to the women they visited on a weekly basis.

*I recommend (the women) to take the supplement, because it is very essential to their lives, especially during pregnancy. Here in the village, our foods mostly do not contain vitamins, it is only millet porridge. If they can be taking the supplement as well, it will help in building their bodies, including the fetus.*

*(Health assistant, IFA group, Phase 1, ID#104)*

### 3.7. Change over the Course of Pregnancy (Longitudinal Analysis)

The collection of longitudinal data (two phases of data collection separated by 4 months) through re-interviews of women who had been early in their pregnancy in the first phase allowed for an understanding of how perceptions of the supplements evolved over the course of the pregnancy. 

Women in their third trimester reported that many of their pregnancy illnesses and symptoms (e.g., nausea, food sensitivities, weakness, headache, body pains) had subsided over time. As a result, the supplement became easier to consume. The same facilitating factors and barriers found earlier were still present, but self-reported adherence increased over time because women had “gotten used to” the supplement and some of their nausea and other symptoms had subsided.

Before, when I started consuming the supplement during early pregnancy, I don’t like it, but as time goes on I started to like it, and now I don’t want to stop consuming the supplement ... The supplement I used to consume before is not sweet like the one I am consuming now that the pregnancy has become matured.

*(Pregnant woman, MQ-LNS group, Phase 2, ID#85)*

Continued efforts by the health workers and other study staff to raise awareness about the supplements’ benefits and to dispel negative rumors, as well as the ability to see other participants in the community deliver healthy babies, appeared to decrease women’s concerns over time. Repeated interviews with the same women in Phase 1 and Phase 2 captured these changes, as shown in the paired quotes below.

What scares me about the supplement is that some women told me that consuming the Plumpy’Nut (MQ-LNS supplement) makes the baby to be big inside the womb to the extent that she cannot deliver the baby by herself that she has to undergo Cesarean section. This is what scares the pregnant women about consuming the Plumpy’Nut because no woman would want to undergo that Cesarean section.

*(Pregnant woman, MQ-LNS group, Phase 1, ID#59)*

When you have a healthy pregnancy … I feel strength and much movement of the fetus. I hear people saying that the baby will be of moderate weight with good complexion. And this is my wish as well … Big or small (baby), it is all from God, and they will all pass through the same passage. You may see a mother who gave birth to a big baby without suffering, while another suffers from delivering a small baby … Most of the women who take this supplement, even if I did not ask them, I know they will have a healthy, mature, and moderate weight baby.

*(Pregnant woman, MQ-LNS group, Phase 2, ID#59)*

### 3.8. Household Spot Checks

A quantitative measure of supplement utilization was collected through unannounced household spot checks. This allowed for triangulation of qualitative results, including reported change over time, as spot checks were conducted during both phases. Supplement consumption was considered to be “correct” if the number of supplements on hand was within 2 days’ worth of the expected number based on time elapsed since last distribution.

**Figure 1:**
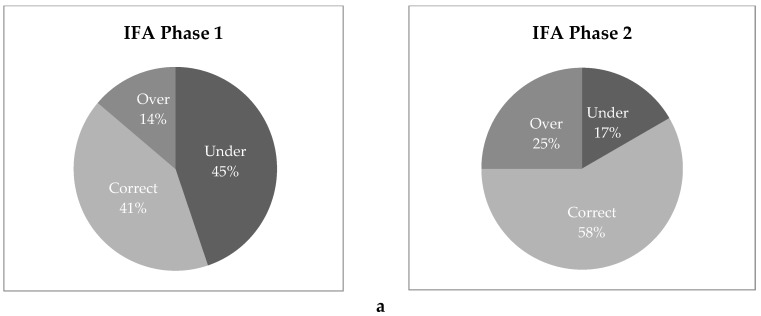

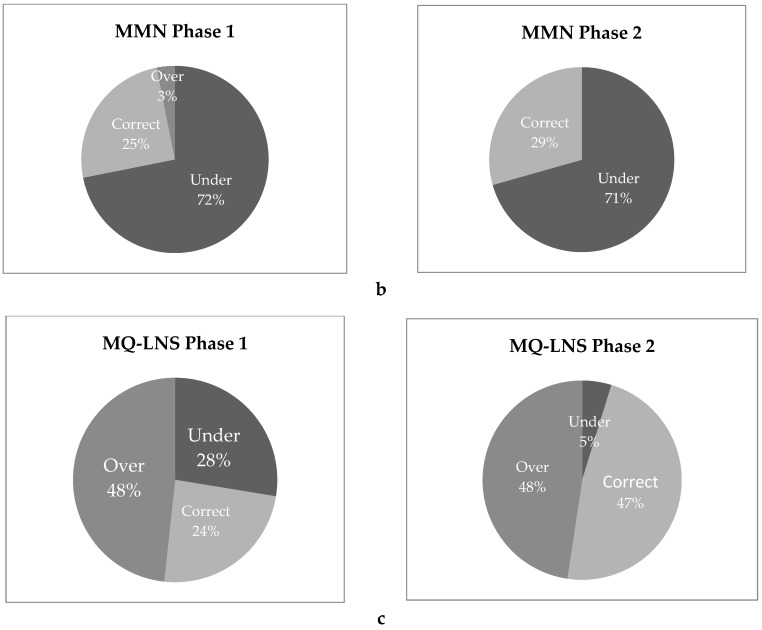
Spot check measure of supplement adherence by study arm and data collection phase: (**a**) IFA, (**b**) MMN, (**c**) MQ-LNS. Note: Correct supplement consumption is defined as having within +/− 2 days’ supply of the expected number of supplements on hand at the time of the spot check.

Adherence varied significantly across the three supplement types. IFA had the highest percentage of correct consumption (46% on average across both phases of data collection), while MMN tended strongly toward under-consumption (71%) and MQ-LNS tended to be over-consumed (48%). Figure 1 shows the spot check results by supplement type and by phase. Correct supplement consumption increased over time for all three supplement types. Although women of all gestational ages were included in Phase 1, only women in late gestation were included in Phase 2, so this data suggests that participants tended to improve their utilization later in pregnancy.

## 4. Discussion

The three nutrient supplements used in this study appeared to be well accepted by the participants, their household members, and their communities. Participants attributed a wide range of benefits to the supplements that were relatively consistent across all three study arms. During pregnancy, the most valued benefits were increased appetite to consume an adequate diet, increased strength to do household chores, and prevention of disease. The supplements were also believed to improve the chances of a safe and uncomplicated delivery of a healthy, well-nourished baby. Utilization, assessed through household spot checks, suggested adherence may not have been consistently correct but was most likely with IFA, while MMN tended to be under-consumed and MQ-LNS over-consumed. Reported acceptability of all three supplement types increased among women later in their pregnancies (Phase 2 interviews), seemingly due to decreased morning sickness and nausea symptoms, as well as participants “getting used to” the supplements.

There were several barriers identified that may have reduced appropriate utilization. For both IFA and MMN, participants strongly felt that the supplements should not be taken on an empty stomach (both due to messaging from study staff and a general belief that this is true for all medications), which led to skipping doses, particularly during fasting for Ramadan and on days where the household did not have food available. For MMN, the large size of the capsule made it difficult to swallow, although many women adopted the alternative approach of opening the capsule and mixing the powder with food or water. For MQ-LNS, sharing of the supplement with household members, particularly small children, was a significant factor. Some women reported hiding the supplement in a secure place, and consuming it only when alone, in order to address this issue. Understanding these barriers and the coping mechanisms developed by participants is important for developing methods to increase adherence in future programs.

The supplementation program was perceived positively overall, particularly due to the high level of trust in medical providers in this study setting. In addition to women’s approval, the study had a high level of support among the men of the community. This is important given the role of husbands as the chief household decision-maker, whose permission was necessary for women to take part. However, some participants and community members remained wary due to prevalent rumors circulating about the study and the supplements. In particular, fear of an invasive vaginal exam (possibly to be carried out by a male doctor) and exaggerated descriptions of up to a liter of blood being drawn led some women to avoid antenatal care and the study enrollment visits. The supplements themselves were feared to cause babies to grow too big, leading to the need for delivery by Cesarean section. Although many participants dismissed this concern, saying that Cesarean sections had existed before the supplement program began and that the size of babies was “God’s will,” it is possible that these rumors played some role in reduced utilization.

The quantitative data from the household spot checks supported the conclusion that the various barriers cited above led to decreased adherence. IFA had the highest percentage of “correct” consumption in the spot checks, but still tended toward under-consumption, likely due to the more generalized fears about blood loss in childbirth and excessively big babies. MMN tended strongly to under-consumption, in accordance with the fact that women found the large capsules difficult to consume. MQ-LNS was most frequently over-consumed, likely due to its perception as a food substitute. Some women reported eating more than one per day if they felt hungry, or sharing with children and other household members. The percentage of correct consumption increased for all three supplement types from Phase 1 to Phase 2, suggesting an improvement in adherence with later gestational age.

The salient themes from this study are consistent with previous research on prenatal supplement acceptability and adherence in West Africa. Past studies have found a high level of utilization, particularly when supplements are provided free of charge [14] and women receive adequate counseling from healthcare providers [14,18]. Comparisons between supplement types, however, have shown varying results. In Mali, adherence to MMN was found to be significantly higher than to IFA [18]; however, in this case, both supplements were delivered in a single daily pill (unlike the present study, in which the four large MMN capsules per day may have acted as a deterrent). In a multi-site study of LNS where comparison groups received either MMN or IFA, adherence to IFA/MMN was significantly higher than LNS in Ghana, while all three were equivalent in Malawi [22]. Sharing of LNS products among household members has been documented in the literature [33]. Barriers to supplement adherence including side effects, organoleptic properties, and pregnancy-related nausea have been reported elsewhere [18,22], but the fears of childbirth complications—which were highly prevalent among this population in Niger—do not appear to be discussed in other prenatal supplementation studies.

One notable strength of the present study is the longitudinal, mixed-methods design, which to our knowledge has been use only once elsewhere [22]. This combination of unannounced spot checks, interviews, and focus group discussions allowed for a unique extension of qualitative data on self-reported supplement consumption behaviors. In addition, few previous studies have examined social and familial factors influencing women’s supplementation practices [17,22], and no study was identified that interviewed husbands and mothers-in-law to directly gather their perceptions. (One recent retrospective study interviewed husbands and wives together about past experiences with prenatal supplementation [34].) Another strength is the criterion-based purposive sampling strategy, which allowed strategic gathering of information from study participants based on personal and program characteristics (e.g., gestational age) for comparison across groups. However, this study has several limitations. Because the interviewers were known by participants to be study employees, it is likely that participants were less willing to share negative feedback or admit that they were not fully adhering to supplementation. Social desirability bias may have also played a role in participants’ unwillingness to report non-adherence [35]. However, women did seem willing to report general rumors and issues in the community, as well as problems encountered by “other women” they knew. Finally, this study was not intended to quantitatively assess compliance; however, compliance has been assessed in the full trial population (forthcoming).

## 5. Conclusions

Despite a stated high level of acceptance and enthusiasm for the supplements among participants and their household members, it is clear that certain fears, side effects, and organoleptic factors impacted utilization. Appropriate messaging to overcome these barriers should be an essential part of any future program that includes prenatal supplementation. For example, addressing rumors about antenatal care and childbirth complications may help to dispel misconceptions and build on the already high level of trust in the medical system; sharing alternative ways of consuming the supplements that other women have found helpful may increase adherence; and emphasizing that supplements can be consumed on an empty stomach if necessary may decrease skipping in an environment of high food insecurity and periodic religious fasting. Husbands, as the key decision-makers in the family setting, should be engaged along with women in these communication efforts.

This study indicates the continued importance of formative and implementation research to understand factors influencing the beliefs and behaviors of pregnant women, as well as household and community members and health staff around them. In order to improve nutritional status and pregnancy outcomes, supplementation programs should take these factors into account to ensure the intervention is well accepted and taken up by participants.

## Figures and Tables

**Table 1:** Qualitative data collection by participant type, study arm, and phase.

	Phase 1 Individual Interviews			Phase 1 Focus Groups			Phase 1 Spot Checks			Phase 2 Individual Interviews			Phase 2 Focus Groups			Phase 2 Spot Checks		
	IFA	MMN	LNS	IFA	MMN	LNS	IFA	MMN	LNS	IFA	MMN	LNS	IFA	MMN	LNS	IFA	MMN	LNS
Pregnant women (<20 weeks gestation)	14	14	12	2	3	7	9	8	10									
Pregnant women (≥20 weeks gestation)	14	16	14	1	2	2	20	24	19	12	13	14	2	3	7	12	17	21
Husbands	7	6	5															
In-laws	2	4	3															
Health assistants				4	2	3												
Study midwives	1	1	1															

IFA: iron and folic acid; MMN: multiple micronutrient; LNS: lipid-based nutrient supplements.

**Table 2:** Summary of main themes from qualitative data.

	**Typical Consumption**					
	Consume only after eating food	Consume with water	Consume with other food	Open capsule before consumption	Overconsume when hungry	
IFA	✔	✔✔	✔			
MMN	✔✔✔	✔	✔✔	✔✔		
MQ-LNS			✔		✔	
	**Perceived Benefits**					
	Increases appetite	Increases strength	Increases blood volume	Improved health of mother and baby	Promotes safe delivery of a healthy child	
IFA	✔✔✔	✔✔	✔✔✔	✔✔✔	✔	
MMN	✔✔✔	✔✔✔	✔✔	✔✔✔	✔	
MQ-LNS	✔✔✔	✔✔✔	✔✔	✔✔	✔	
	**Side Effects**					
	Bad odor	Too large for consumption	“Excess blood” in the body	Bad taste		
IFA	✔	✔	✔✔			
MMN	✔✔	✔✔				
MQ-LNS				✔		
	**Facilitating Factors**					
	Trust in medical system	Positive perception of parent study	Saw healthy babies delivered in community			
IFA	✔✔	✔✔	✔			
MMN	✔✔	✔✔	✔			
MQ-LNS	✔✔	✔✔	✔			
	**Barriers to Consumption**					
	Rumors about parent study	Fear of delivering large baby	Fear of Bleeding/Complications during delivery	Limited access to food to consume supplement	Overconsume when hungry	Share with children or sell for money
IFA	✔✔✔	✔✔✔	✔✔	✔		
MMN	✔✔✔	✔✔	✔	✔✔		
MQ-LNS	✔✔✔	✔✔			✔	✔✔

Check marks is based on the relative frequency with which a theme was coded in transcripts from that study arm (✔ = least frequent, ✔✔ = intermediate frequency, ✔✔✔ = most frequent).

## Supplementary Materials

The following are available online at http://www.mdpi.com/2072-6643/10/8/1073/s1, Table S1: Nutritional composition of study supplements.
