# Supplementary material for: Acceptability and Utilization of Three Nutritional Supplements during Pregnancy: Findings from a Longitudinal, Mixed-Methods Study in Niger
Source: Nutrients. 2018 Aug 12;10(8):1073. doi: 10.3390/nu10081073 (PMC6115835; doi:10.3390/nu10081073)
Supplement: Supplementary file 1 [file nutrients-10-01073-s001.pdf]

Table S1: Nutritional composition of study supplements

	US RDA/AI for pregnant women	IFA	MMN	MQ-LNS (40g)
Energy (kcal)	2500	---	---	237
Proteins (g)	50	---	---	5.2
Milk proteins (g)	---	---	---	1.7
Lipids (g)	---	---	---	20
LA (*Linoleic Acid*) (g)	---	---	---	6.9
ALA (*α-Linolenic Acid*) (g)	---	---	---	1.16
Calcium (mg)	1000	---	559	559
Phosphorus (*free*) (mg)	700	---	400	400
Potassium (mg)	4700	---	400	400
Magnesium (mg)	350	---	130	130
Zinc (mg)	11	---	30	30
Copper (mg)	1	---	4	4
Iron (mg)	27	60	30	30
Manganese (mg)	2	---	2.6	2.6
Iodine (µg)	220	---	250	250
Selenium (µg)	60	---	130	130
Vitamin A (µg)	800	---	800	800
Vitamin B_1 (mg)	1.4	---	2.8	2.8
Vitamin B_2 (mg)	1.4	---	2.8	2.8
Niacin B_3 (mg)	18	---	36	36
Pantothenic acid - B_5(mg)	6	---	7	7
Vitamin B_6 (mg)	1.9	---	3.8	3.8
Vitamin B_{12} (µg)	2.6	---	5.2	5.2
Folic acid (µg)	600	400	400	400
Vitamin C (mg)	85	---	100	100
Vitamin D_3 (µg)	15	---	15	15
Vitamin E (mg)	15	---	20	20
Vitamin K_1 (µg)	90	---	45	45

RDA / AI = recommended dietary allowance / adequate intake
IFA = Iron and folic acid
MMN = Multiple micronutrients
MQ-LNS = Medium-quantity lipid-based nutrient supplement
